# SARS-CoV-2 Epidemiology and COVID-19 mRNA Vaccine Effectiveness Among Infants and Children Aged 6 Months–4 Years — New Vaccine Surveillance Network, United States, July 2022–September 2023

**DOI:** 10.15585/mmwr.mm7248a2

**Published:** 2023-12-01

**Authors:** Ayzsa Tannis, Janet A. Englund, Ariana Perez, Elizabeth J. Harker, Mary Allen Staat, Elizabeth P. Schlaudecker, Natasha B. Halasa, Laura S. Stewart, John V. Williams, Marian G. Michaels, Rangaraj Selvarangan, Jennifer E. Schuster, Leila C. Sahni, Julie A. Boom, Geoffrey A. Weinberg, Peter G. Szilagyi, Benjamin R. Clopper, Yingtao Zhou, Meredith L. McMorrow, Eileen J. Klein, Heidi L. Moline

**Affiliations:** ^1^Coronavirus and Other Respiratory Viruses Division, National Center for Immunization and Respiratory Diseases, CDC; ^2^Eagle Health Analytics, LLC, San Antonio, Texas; ^3^Department of Pediatrics, Seattle Children’s Hospital, Seattle, Washington; ^4^Influenza Division, National Center for Immunization and Respiratory Diseases, CDC; ^5^Division of Infectious Diseases, Cincinnati Children’s Hospital Medical Center, Cincinnati, Ohio; ^6^Department of Pediatrics, University of Cincinnati College of Medicine, Cincinnati, Ohio; ^7^Department of Pediatrics, Vanderbilt University Medical Center, Nashville, Tennessee; ^8^UPMC Children’s Hospital of Pittsburgh, Pittsburgh, Pennsylvania; ^9^Department of Pediatrics, University of Pittsburgh School of Medicine, Pittsburgh, Pennsylvania; ^10^Department of Pathology and Laboratory Medicine, Children’s Mercy Hospital, Kansas City, Missouri; ^11^Texas Children’s Hospital, Houston, Texas; ^12^Baylor College of Medicine, Houston, Texas; ^13^Department of Pediatrics, University of Rochester Medical Center, Rochester, New York; ^14^University of California at Los Angeles, Los Angeles, California; ^15^Maximus, Inc., Atlanta, Georgia.

**Summary**

**What is already known about this topic?**

SARS-CoV-2 infection in young children is often mild or asymptomatic; however, some children are at risk for severe disease. In June 2022, original monovalent COVID-19 mRNA vaccines were recommended for infants and children aged 6 months–4 years.

**What is added by this report?**

Among vaccine-eligible children aged <5 years hospitalized or seeking care in emergency departments for acute respiratory illness during July 2022–September 2023, 86% had not received any COVID-19 vaccine. Despite low vaccination coverage, only 5% of children received a positive SARS-CoV-2 test result. Receipt of ≥2 COVID-19 mRNA vaccine doses was 40% effective (95% CI = 8%–60%) in preventing emergency department visits and hospitalization.

**What are the implications for public health practice?**

These findings support existing recommendations for COVID-19 vaccination of young children to reduce COVID-19–associated emergency department visits and hospitalization.

## Abstract

SARS-CoV-2 infection in young children is often mild or asymptomatic; however, some children are at risk for severe disease. Data describing the protective effectiveness of COVID-19 mRNA vaccines against COVID-19–associated emergency department (ED) visits and hospitalization in this population are limited. Data from the New Vaccine Surveillance Network, a prospective population-based surveillance system, were used to estimate vaccine effectiveness using a test-negative, case-control design and describe the epidemiology of SARS-CoV-2 in infants and children aged 6 months–4 years during July 1, 2022–September 30, 2023. Among 7,434 children included, 5% received a positive SARS-CoV-2 test result, and 95% received a negative test result; 86% were unvaccinated, 4% had received 1 dose of any vaccine product, and 10% had received ≥2 doses. When compared with receipt of no vaccines among children, receipt of ≥2 COVID-19 mRNA vaccine doses was 40% effective (95% CI = 8%–60%) in preventing ED visits and hospitalization. These findings support existing recommendations for COVID-19 vaccination of young children to reduce COVID-19–associated ED visits and hospitalization.

## Introduction

SARS-CoV-2 infection in young children and adolescents commonly manifests as a mild or asymptomatic illness; however, some children are at risk for severe disease, including those with certain chronic conditions (*1*,*2*). COVID-19 mRNA vaccines were recommended for children aged ≥5 years in November 2021, and for infants and children aged 6 months–4 years in June 2022, with further authorizations for bivalent mRNA vaccines during December 2022–April 2023 (*3*). Vaccination coverage in this population remains markedly lower than that in the adult population, and complete primary series COVID-19 mRNA vaccination coverage in young children has been approximately 5% nationwide since January 2023.^†^ As such, vaccine effectiveness (VE) estimates in infants and children aged 6 months–4 years are limited (*4*,*5*). Despite low coverage in this age group, COVID-19–associated hospitalization rates among infants and children aged 6 months–4 years has remained low.^§^ This analysis assessed the effectiveness of COVID-19 mRNA vaccines in protecting against COVID-19–associated emergency department (ED) visits and hospitalization during the first year of authorization of vaccination for infants and children aged 6 months–4 years, a period when several Omicron sublineages were circulating.^¶^

## Methods

### Data Collection

The New Vaccine Surveillance Network (NVSN) conducts population-based, prospective surveillance for acute respiratory illness (ARI) in children at seven pediatric medical centers.** During July 1, 2022–September 30, 2023, infants and children aged 6 months–4 years hospitalized or seeking care in EDs for ARI were eligible for enrollment.^††^ Demographic, clinical, and vaccination data were systematically collected through parent or guardian interview and medical chart abstraction. Respiratory specimens were collected and tested for SARS-CoV-2 and seven other respiratory viruses^§§^ using real-time reverse transcription–polymerase chain reaction. COVID-19 vaccination status was ascertained through state immunization information systems and verified, if necessary, by reviewing health care provider records.^¶¶^

### Data Analysis

COVID-19 VE to prevent COVID-19–associated ED visits and hospitalization among children with ARI was estimated using a test-negative, case-control design. Case-patients were children with ARI and who received a positive SARS-CoV-2 test result. Control-patients were children with ARI and who received a negative SARS-CoV-2 test result. Children were included in the analysis if they had a verified vaccination status including 1) zero doses of any COVID-19 vaccine product (unvaccinated), 2) 1 dose of any COVID-19 vaccine product (1 dose only), or 3) ≥2 doses of any COVID-19 vaccine product (≥2 doses). Children were excluded if they met NVSN exclusion criteria,*** were enrolled <14 days after receipt of a vaccine dose, received an inconclusive SARS-CoV-2 test result, were missing COVID-19 vaccination data, or if receipt of vaccination was unverified. Pearson’s chi-square tests were used to compare demographic and clinical characteristics among case- and control-patients and by vaccination status. VE was estimated using logistic regression models, comparing the odds of receipt of 1 or ≥2 vaccine doses with those with no COVID-19 vaccination between case- and control-patients. Regression models controlled for race, age, calendar time (week of enrollment), and enrollment site. VE was calculated as (1 – adjusted odds ratio) x 100%; estimates with nonoverlapping 95% CIs were considered statistically significant. SAS (version 9.4; SAS Institute) was used to conduct all analyses. This activity was reviewed by CDC, deemed not research, and was conducted consistent with applicable federal law and CDC policy.^†††^

## Results

### Differences Between Case-Patients and Control-Patients

During July 1, 2022–September 30, 2023, among 7,434 infants and children aged 6 months–4 years with ARI enrolled in ED or hospital settings, 387 (5.0%) received a positive SARS-CoV-2 test result, and 7,047 (95.0%) received a negative test result (Table 1). Case-patients were significantly younger than were control-patients (median age 15 months versus 22 months, respectively). There was no difference in median length of stay (2 days), sex, race and ethnicity, insurance status, history of prematurity, or underlying medical conditions between case- and control-patients. Case-patients were less likely to receive supplemental oxygen and high-flow nasal cannula respiratory support than were control-patients; however, there was no difference between case- and control-patients in the proportion who received mechanical ventilation or were admitted to an intensive care unit. Two case-patients (0.5%) were intubated, none received extracorporeal membrane oxygenation, and none died, compared with 69 (1.0%), three (0.9%), and three (0.1%) control-patients, respectively. Other respiratory viruses were detected in 140 (36.2%) case-patients; rhinoviruses/enteroviruses (RV/EV) accounted for one half of these detections, and respiratory syncytial virus accounted for 21.4%. Among control-patients, RV/EV and respiratory syncytial virus also accounted for the majority of detections and were detected in 36.7% and 17.1% of control-patients, respectively.

**TABLE 1:** Characteristics of infants and children* aged 6 months–4 years enrolled in vaccine effectiveness study, by SARS-CoV-2 test result and COVID-19 vaccination status (N = 7,434) — New Vaccine Surveillance Network, United States, July 2022–September 2023

Characteristic	Overall (column %) N = 7,434	SARS-CoV-2 test result (column %)			Vaccination status (row %)			
		Positive (case-patients) n = 387	Negative (control-patients) n = 7,047	p-value^†^	Unvaccinated n = 6,377	1 dose only n = 281	≥2 doses n = 776	p-value^†,§^
**Highest level of care**								
ED	**4,026 (54.2)**	247 (63.8)	3,779 (53.6)	<0.001	3,557 (88.4)	127 (3.2)	342 (8.5)	<0.001
Inpatient	**3,408 (45.8)**	140 (36.2)	3,268 (46.4)		2,820 (82.7)	154 (4.5)	434 (12.7)	
**Study site**								
Cincinnati, Ohio	**1,328 (17.9)**	55 (14.2)	1,273 (18.1)	0.013	1,251 (94.2)	19 (1.4)	58 (4.4)	<0.001
Houston, Texas	**1,048 (14.1)**	67 (17.3)	981 (13.9)		943 (90.0)	32 (3.1)	73 (7.0)	
Kansas City, Missouri	**845 (11.4)**	39 (10.1)	806 (11.4)		792 (93.7)	20 (2.4)	33 (3.9)	
Nashville, Tennessee	**1,121 (15.1)**	56 (14.5)	1,065 (15.1)		1,004 (89.6)	43 (3.8)	74 (6.6)	
Pittsburgh, Pennsylvania	**1,239 (16.7)**	50 (12.9)	1,189 (16.9)		1,066 (86.0)	44 (3.6)	129 (10.4)	
Rochester, New York	**570 (7.7)**	37 (9.6)	533 (7.6)		499 (87.5)	20 (3.5)	51 (8.9)	
Seattle, Washington	**1,283 (17.3)**	83 (21.4)	1,200 (17.0)		822 (64.1)	103 (8.0)	358 (27.9)	
**Age, mos**								
Median (IQR)	**22 (12.0–37.0)**	15 (9.0–29.0)	22 (13.0–38.0)	<0.001	21 (12.0–37.0)	25 (13.0–38.0)	27 (17.0–40.5)	<0.001
6–11	**1,640 (22.1)**	146 (37.7)	1,494 (21.2)	<0.001	1,536 (93.7)	54 (3.3)	50 (3.0)	<0.001
12–23	**2,329 (31.3)**	122 (31.5)	2,207 (31.3)		1,970 (84.6)	80 (3.4)	279 (12.0)	
24–59	**3,465 (46.6)**	119 (30.7)	3,346 (47.5)		2,871 (82.9)	147 (4.2)	447 (12.9)	
**Sex**								
Female	**3,214 (43.2)**	170 (43.9)	3,044 (43.2)	0.777	2,765 (86.0)	125 (3.9)	324 (10.1)	0.394
Male	**4,220 (56.8)**	217 (56.1)	4,003 (56.8)		3,612 (85.6)	156 (3.7)	452 (10.7)	
**Race and ethnicity**								
Black or African American, NH	**2,277 (30.6)**	98 (25.3)	2,179 (30.9)	0.055	2,169 (95.3)	50 (2.2)	58 (2.5)	<0.001
White, NH	**2,218 (29.8)**	127 (32.8)	2,091 (29.7)		1,707 (77.0)	90 (4.1)	421 (19.0)	
Hispanic or Latino	**1,938 (26.1)**	112 (28.9)	1,826 (25.9)		1,741 (89.8)	73 (3.8)	124 (6.4)	
Other, NH	**857 (11.5)**	47 (12.1)	810 (11.5)		638 (74.4)	60 (7.0)	159 (18.6)	
Unknown	**144 (1.9)**	3 (0.8)	141 (2.0)		122 (84.7)	8 (5.6)	14 (9.7)	
**Insurance status**								
Private	**2,085 (28.0)**	106 (27.4)	1,979 (28.1)	0.645	1,426 (68.4)	132 (6.3)	527 (25.3)	<0.001
Public	**4,726 (63.6)**	254 (65.6)	4,472 (63.5)		4,394 (93.0)	126 (2.7)	206 (4.4)	
Public and private	**130 (1.7)**	6 (1.6)	124 (1.8)		107 (82.3)	8 (6.2)	15 (11.5)	
Self-pay (none)	**227 (3.1)**	7 (1.8)	220 (3.1)		213 (93.8)	6 (2.6)	8 (3.5)	
Unknown	**266 (3.6)**	14 (3.6)	252 (3.6)		237 (89.1)	9 (3.4)	20 (7.5)	
**Median no. of days since last vaccine dose (IQR)**	**86 (46.0–160.0)**	76 (31.0–171.0)	86 (47.0–160.0)	0.021	NA	71 (31.0–128.0)	93 (51.0–171.5)	0.083
**Prematurity** ^¶^	**781 (20.1)**	44 (17.0)	737 (20.3)	0.194	705 (90.3)	20 (2.6)	56 (7.2)	0.206
**Underlying conditions**								
One or more**	**1,916 (26.4)**	104 (27.5)	1,812 (26.3)	0.608	1,576 (82.3)	90 (4.7)	250 (13.0)	<0.001
Cardiovascular condition^††^	**336 (4.6)**	27 (7.1)	309 (4.5)	0.017	282 (83.9)	12 (3.6)	42 (12.5)	0.261
Immunocompromised^§§^	**101 (1.4)**	12 (3.2)	89 (1.3)	0.002	90 (89.1)	3 (3.0)	8 (7.9)	0.359
Neurologic condition^¶¶^	**373 (5.1)**	25 (6.6)	348 (5.1)	0.181	321 (86.1)	16 (4.3)	36 (9.7)	0.550
Respiratory condition***	**1,101 (15.2)**	40 (10.6)	1,061 (15.4)	0.011	880 (79.9)	53 (4.8)	168 (15.3)	<0.001
Other condition	**674 (26.7)**	32 (23.9)	642 (26.8)	0.456	560 (83.1)	30 (4.5)	84 (12.5)	0.750
**Respiratory support**								
Supplemental oxygen	**2,206 (55.4)**	63 (29.3)	2,143 (56.8)	<0.001	1,816 (82.3)	99 (4.5)	291 (13.2)	<0.001
Nasal cannula or blowby	**1,025 (77.3)**	32 (88.9)	993 (77.0)	0.092	803 (78.3)	46 (4.5)	176 (17.2)	0.050
High-flow nasal cannula	**472 (35.6)**	4 (11.4)	468 (36.2)	0.002	396 (83.9)	23 (4.9)	53 (11.2)	<0.001
CPAP or BiPAP therapy	**138 (10.5)**	4 (11.1)	134 (10.4)	0.896	115 (83.3)	4 (2.9)	19 (13.8)	0.382
Intubation	**71 (1.0)**	2 (0.5)	69 (1.0)	0.363	62 (87.3)	4 (5.6)	5 (7.0)	0.347
ECMO^†††^	**3 (0.9)**	0 (—)	3 (0.9)	0.789	3 (100.0)	0 (—)	0 (—)	0.488
**Received intensive care** ^†††^	**347 (17.0)**	8 (10.5)	339 (17.2)	0.126	289 (83.3)	12 (3.5)	46 (13.3)	0.271
**Length of stay, days^†††^**								
Median (IQR)	**2 (1.0–3.0)**	2 (1.0–3.0)	2 (1.0–3.0)	0.350	2 (1.0–3.0)	2 (1.0–3.0)	1 (1.0–3.0)	0.748
0–1	**1,005 (49.2)**	34 (44.7)	971 (49.4)	0.281	787 (78.3)	53 (5.3)	165 (16.4)	0.058
2	**475 (23.3)**	23 (30.3)	452 (23.0)		398 (83.8)	21 (4.4)	56 (11.8)	
3–4	**330 (16.2)**	14 (18.4)	316 (16.1)		260 (78.8)	15 (4.5)	55 (16.7)	
≥5	**232 (11.4)**	5 (6.6)	227 (11.5)		195 (84.1)	7 (3.0)	30 (12.9)	
**Death**	**3 (0.1)**	0 (—)	3 (0.1)	0.641	2 (66.7)	1 (33.3)	0 (—)	0.692
**Viral detections^§§§^**								
One or more viruses	**5,560 (74.8)**	387 (100.0)	5,173 (73.4)	<0.001	4,701 (84.6)	222 (4.0)	637 (11.5)	<0.001
RV/EV	**2,720 (36.6)**	70 (18.1)	2,650 (37.6)	<0.001	2,266 (83.3)	115 (4.2)	339 (12.5)	<0.001
RSV	**1,236 (16.6)**	30 (7.8)	1,206 (17.1)	<0.001	1,043 (84.4)	57 (4.6)	136 (11.0)	0.642
Adenovirus	**795 (10.7)**	17 (4.4)	778 (11.0)	<0.001	656 (82.5)	43 (5.4)	96 (12.1)	0.161
PIV	**747 (10.0)**	15 (3.9)	732 (10.4)	<0.001	647 (86.6)	21 (2.8)	79 (10.6)	0.461
HMPV	**534 (7.2)**	14 (3.6)	520 (7.4)	0.023	451 (84.5)	21 (3.9)	62 (11.6)	0.480
EV-D68	**277 (3.7)**	9 (2.3)	268 (3.8)	<0.001	246 (88.8)	9 (3.2)	22 (7.9)	0.451
HCoV	**188 (2.5)**	7 (1.8)	181 (2.6)	0.508	143 (76.1)	10 (5.3)	35 (18.6)	0.001
Influenza	**157 (2.1)**	4 (1.0)	153 (2.2)	0.033	143 (91.1)	4 (2.5)	10 (6.4)	0.098
SARS-CoV-2 codetection^¶¶¶^	**140 (1.9)**	140 (36.2)	NA	—	126 (90.0)	3 (2.1)	11 (7.9)	0.284
RV/EV^¶¶¶^	**70 (50.0)**	70 (50.0)			62 (88.6)	3 (4.3)	5 (7.1)	0.811
RSV^¶¶¶^	**30 (21.4)**	30 (21.4)			28 (93.3)	0 (—)	2 (6.7)	0.756
Adenovirus^¶¶¶^	**17 (12.1)**	17 (12.1)			17 (100.0)	0 (—)	0 (—)	0.405
PIV^¶¶¶^	**15 (10.7)**	15 (10.7)			12 (80.0)	0 (—)	3 (20.0)	0.189
HMPV^¶¶¶^	**14 (10.0)**	14 (10.0)			13 (92.9)	0 (—)	1 (7.1)	0.948
EV-D68^¶¶¶^	**9 (6.4)**	9 (6.4)			8 (88.9)	0 (—)	1 (11.1)	0.429
HCoV^¶¶¶^	**7 (5.0)**	7 (5.0)			6 (85.7)	0 (—)	1 (14.3)	0.793
Influenza^¶¶¶^	**4 (2.9)**	4 (2.9)			4 (100.0)	0 (—)	0 (—)	0.575

**Abbreviations:** BiPAP = bilevel positive airway pressure; CPAP = continuous positive airway pressure; ECMO = extracorporeal membrane oxygenation; ED = emergency department; EV-D68 = enterovirus D68; HCoV = human coronaviruses; HMPV = human metapneumovirus; NA = not applicable; NH = non-Hispanic; PIV = parainfluenza viruses 1–4; RSV = respiratory syncytial virus; RV/EV = rhinovirus/enterovirus.

* Restricted to children enrolled in inpatient and ED clinical settings.

^†^ p-value refers to results of Pearson’s chi-square comparison.

^§^ p-value measuring difference between unvaccinated children and children receiving ≥2 vaccine doses.

^¶^ Gestational age <37 weeks, restricted to infants and children aged <2 years.

** Underlying medical conditions include congenital heart malformation or other heart condition, transplant recipient, cancer, sickle cell anemia, cerebral palsy, seizure disorder or other neurologic or neuromuscular disorder, asthma, reactive airway disease, cystic fibrosis, bronchopulmonary dysplasia, chronic lung disease of prematurity or other chronic lung condition, kidney disease, Down syndrome or other genetic or metabolic disorder, blood disorders, liver disease, diabetes, chronic endocrine condition, chronic gastrointestinal disease, and other developmental disabilities.

^††^ Congenital heart malformation or other heart condition.

^§§^ Immune condition, transplant recipient (peripheral blood stem cells, bone marrow, cord blood, or organ), cancer, and sickle cell anemia.

^¶¶^ Cerebral palsy, seizure disorder, or other neurologic or neuromuscular disorder.

*** Asthma, reactive airway disease, cystic fibrosis, bronchopulmonary dysplasia, chronic lung disease of prematurity, or other chronic lung condition.

^†††^ Among hospitalized children only.

^§§§^ Among all children.

^¶¶¶^ Among children who received a positive SARS-CoV-2 test result only.

### Sociodemographic Characteristics by Vaccination Coverage Status

During this period, 86.0% of infants and children aged 6 months–4 years with ARI had not received any COVID-19 vaccine doses; 2-dose vaccination coverage varied significantly geographically, from 3.9% to 27.9% across NVSN sites. Children receiving ≥2 COVID-19 vaccine doses were more likely to be 1) from Seattle (27.9%), 2) non-Hispanic White (White) or non-Hispanic other race (37.6%), and 3) have private insurance (25.3%). Overall, 2-dose vaccination coverage was 19.0% among White children and 2.5% among non-Hispanic Black or African American (Black) children. Children who had received ≥2 COVID-19 vaccine doses were older (median age = 27 months) than unvaccinated children (median age = 21 months).

Weekly SARS-CoV-2 detections peaked once during August 31–September 6, 2022, (21) and again during August 27–September 2, 2023 (13) (Figure). Cumulative coverage with ≥2 COVID-19 vaccine doses was 10.4% and with 1 dose was 3.8%.

**FIGURE:**
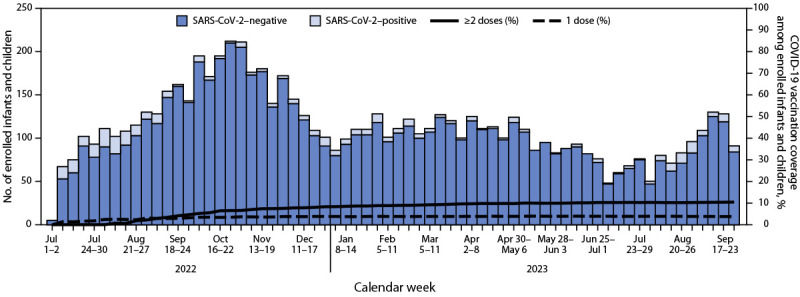
SARS-CoV-2 test results and COVID-19 vaccination coverage among infants and children aged 6 months–4 years evaluated in the emergency department or hospitalized with acute respiratory illness, by week (N = 7,434) — New Vaccine Surveillance Network, United States, July 2022—September 2023

### Vaccine Effectiveness

When compared with no receipt of COVID-19 vaccination among children, the estimated VE of ≥2 COVID-19 mRNA vaccine doses was 40% (95% CI = 8%–60%) for preventing COVID-19–associated ED visits and hospitalization, with a median interval since receipt of last vaccine dose of 93 days (IQR = 51–172 days) (Table 2). VE of 1 mRNA COVID-19 vaccine dose for preventing COVID-19–associated ED visits and hospitalization was 31% (95% CI = –27% to 62%), although the 95% CI included the null value.

**TABLE 2:** COVID-19 vaccine effectiveness among infants and children aged 6 months–4 years evaluated in the emergency department or hospitalized with acute respiratory illness (N = 7,434) — New Vaccine Surveillance Network, United States, July 2022–September 2023*

Vaccination status	No. (%)		Median no. of days since last dose (IQR)	Adjusted VE,^†^ % (95% CI)
	Case-patients (positive SARS-CoV-2 test result) n = 387	Control patients (negative SARS-CoV-2 test result) n = 7,047		
Unvaccinated	348 (90)	6,029 (85)	NA	—
Vaccinated	39 (10)	1,018 (15)	Not calculated	Not calculated
1 dose only	12 (3)	269 (4)	71 (31 to 128)	31 (–27 to 62)
≥2 doses	27 (7)	749 (11)	93 (51 to 172)	40 (8 to 60)^§^

**Abbreviations:** NA = not applicable; VE = vaccine effectiveness.

* Some estimates are imprecise, which might be because of a relatively small number of persons in each level of vaccination or case-patient status. This imprecision indicates the actual VE could be substantially different from the point estimate shown, and estimates should therefore be interpreted with caution. Additional data accrual could increase precision and allow appropriate interpretation.

^†^ VE was estimated by comparing odds of being vaccinated with 1 dose or ≥2 doses among case-patients to the odds of being vaccinated with 1 dose or ≥2 doses among control patients. Calculated as VE = 100 x (1 – odds ratio). Regression models adjusted for race, age, calendar time (week of enrollment), and enrollment site.

^§^ p<0.05.

## Discussion

In this analysis of 7,434 infants and children aged 6 months–4 years with ARI in NVSN, 86.0% had not received any COVID-19 vaccine doses, and clear geographic, age, and racial differences in vaccination coverage were observed: ≥2-dose coverage in Seattle was approximately 2–6 times that of other NVSN sites, which is consistent with high vaccination coverage in this region for other routine childhood vaccines.^§§§^ Compared with White children, Black children were approximately seven times less likely and Hispanic or Latino children were approximately three times less likely to have received ≥2 doses of COVID-19 vaccine, underscoring the continued need to promote access and address vaccine hesitancy (*6*).

Among young children with medically attended ARI, SARS-CoV-2 detections were low, with just 5% of children receiving a positive SARS-CoV-2 test result. Co-detections of other respiratory viruses were present in approximately one third of children who received positive SARS-CoV-2 test results. Systematic testing for multiple respiratory viruses is a strength of NVSN and provides essential information on co-detections that is not possible from isolated SARS-CoV-2 testing. It might be important to account for coinfections in future VE estimates, particularly as more vaccines are introduced for respiratory viruses that could bias pediatric VE estimates.

Receipt of ≥2 COVID-19 mRNA vaccine doses was 40% effective in preventing COVID-19–associated ED visits and hospitalization. Despite low vaccination coverage and the circulation of several Omicron subvariants, COVID-19–associated ED visits and hospitalization among children with ARI enrolled in NVSN were rare, suggesting most children in this age group experience mild illness from these subvariants or have immune protection from previous SARS-CoV-2 exposure (*7*). These findings indicate that COVID-19 mRNA vaccines are protective and are consistent with other VE estimates for this age group, ranging from 29% for 2-dose Moderna coverage to 43% for 3-dose Pfizer-BioNTech coverage (*5*); however, low vaccination coverage and low incidence of medically attended COVID-19 limit precision in these VE estimates.

### Limitations

The findings in this report are subject to at least five limitations. First, seroprevalence of infection-induced SARS-CoV-2 antibodies in children and adolescents has increased over time, which might affect VE estimates and assessment of severe outcomes, as more children have immunity from previous SARS-CoV-2 infection (*8*). Second, low vaccination coverage might indicate that vaccinated children are systematically different from unvaccinated children. For example, children with underlying medical conditions might be more likely to be vaccinated and, because of their underlying conditions, more likely to be hospitalized or to need respiratory support, which could bias the observed VE. Third, NVSN data might be subject to enrollment biases that might vary by site, such as number of enrollment days per week and availability of interpreters for non-English speakers. Fourth, low vaccination coverage and disease incidence limit the precision of the point estimates and were too low to analyze data by time since dose or to stratify by setting or product. Finally, Moderna vaccine is administered as a 2-dose primary series whereas Pfizer-BioNTech requires 3 doses, and receipt of ≥2 doses might underestimate the protection afforded by the complete 3-dose Pfizer-BioNTech primary series.

### Implications for Public Health Practice

Limited data are available on the impact of COVID-19 vaccination among infants and children aged 6 months–4 years to help guide vaccination policies. Data from this study are consistent with those from other studies of COVID-19 mRNA VE among young children and might assist medical providers when counseling parents of young children about COVID-19 vaccination (*4*,*5*). The findings in this report support the recommendation for COVID-19 vaccination for all children aged ≥6 months and highlight the importance of completion of a primary series for young children (*3*).

## Footnotes

* These senior authors contributed equally to this report.

†
https://covid.cdc.gov/covid-data-tracker/#vaccination-demographics-trends

§
https://www.cdc.gov/coronavirus/2019-ncov/covidnetdashboard/de/powerbi/dashboard.html

¶
https://www.cdc.gov/museum/timeline/covid19.html#Mid-2022

** Children’s Hospital of Pittsburgh, Pittsburgh, Pennsylvania; Children’s Mercy Hospital, Kansas City, Missouri; Cincinnati Children’s Hospital Medical Center, Cincinnati, Ohio; Golisano Children’s Hospital, Rochester, New York; Seattle Children’s Hospital, Seattle, Washington; Texas Children’s Hospital, Houston, Texas; Vanderbilt University Medical Center, Nashville, Tennessee.

†† ARI is defined as one or more of the following symptoms occurring for <14 days before enrollment encounter: fever, cough, earache, nasal congestion, runny nose, sore throat, vomiting after coughing, wheezing, shortness of breath, rapid or shallow breathing, apnea, apparent life-threatening event, or brief resolved unexplained event.

§§ All children received testing for the following viruses: SARS-CoV-2, rhinovirus/enterovirus, respiratory syncytial virus, human metapneumovirus, enterovirus-D68, parainfluenza viruses, human coronaviruses, and influenza viruses.

¶¶ Primary care provider record verification was required after the expiration of the public health emergency in sites without mandatory reporting of COVID-19 vaccines to state immunization information systems.

*** 1) age ≥18 years, 2) residence outside surveillance area, 3) admitted patient not enrolled or specimen collected ≤48 hours of hospital admission, 4) fever and neutropenia (absolute neutrophil count <500), 5) newborn who never left the hospital, 6) transferred from another hospital admission of >48 hours, 7) known nonrespiratory cause for admission, 8) duration of illness lasting >10 days, or 9) previous encounter <10 days before hospital admission at the same level or higher level of care and not enrolled.

††† 45 C.F.R. part 46, 21 C.F.R. part 56; 42 U.S.C. Sect. 241(d); 5 U.S.C. Sect. 552a; 44 U.S.C. Sect. 3501 et seq.

§§§
https://www.cdc.gov/vaccines/imz-managers/coverage/childvaxview/interactive-reports/index.html
